# Liver metastasis from hepatoid adenocarcinoma of the esophagus mimicking hepatocellular carcinoma

**DOI:** 10.1093/gastro/gov021

**Published:** 2015-06-20

**Authors:** Amir Kashani, Jonathan C. Ellis, Melissa Kahn, Laith H. Jamil

**Affiliations:** 1 Division of Gastroenterology, Cedars-Sinai Medical Center, Los Angeles, CA, USA; 2 Department of Pathology and Laboratory Medicine, Cedars-Sinai Medical Center, Los Angeles, CA, USA

**Keywords:** hepatoid adenocarcinoma, alpha-fetoproteins, hepatocellular carcinoma

## Abstract

Alpha-fetoprotein (AFP)-producing adenocarcinoma, histologically mimicking hepatocellular carcinoma (HCC), is a distinct entity known as hepatoid adenocarcinoma (HAC). Reported cases of HAC arising from the esophagus are extremely rare. Due to common liver metastasis and elevated AFP levels in patients with esophageal HAC, differentiation of HAC with liver metastasis from HCC could be challenging. We describe a case of esophageal HAC that presented with a liver mass showing hepatoid features and elevated serum AFP levels. Initial presentation was suspicious for HCC. Upon further diagnostic work-up, the patient was diagnosed with esophageal HAC with liver metastasis. The distinction between these two entities is particularly important because HAC is more aggressive, and its therapeutic options are very limited.

## Introduction

Alpha-fetoprotein (AFP)-producing adenocarcinoma, mimicking hepatocellular carcinoma (HCC) histology, was first reported in 1970 [1]; this was later termed hepatoid adenocarcinoma (HAC) [2]. Due to common liver metastasis and elevated AFP levels, HAC-associated liver metastasis falls into the list of differential diagnoses for HCC [3]. The distinction between these two entities is crucial because HAC is more aggressive and has limited therapeutic options [3,4]. When an AFP-producing adenocarcinoma with hepatoid features is found in the liver, differentiating this entity from HCC might be more difficult; therefore, an extensive diagnostic work-up for the primary site is highly warranted in this scenario [3]. Herein we report a case of HAC of the esophagus with liver metastasis in which the initial presentation resembled HCC.

## Case presentation

**Figure 1.**
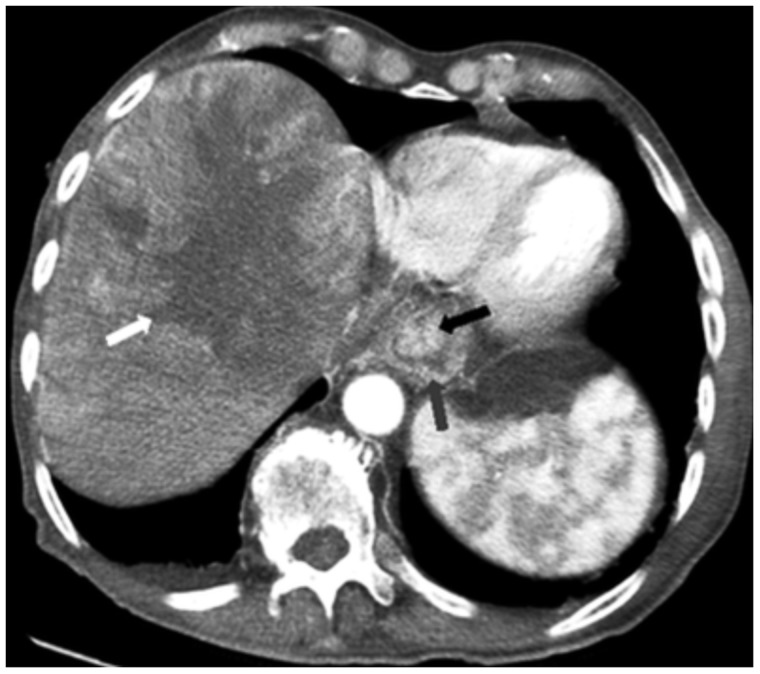
Computed tomography scan reveals a 12.6 × 11.2 cm liver mass with central necrosis and heterogeneous enhancement (white arrow) and esophageal wall thickening (gray arrow) with a large luminal mass (black arrow) almost obstructing the lumen.

**Figure 2.**
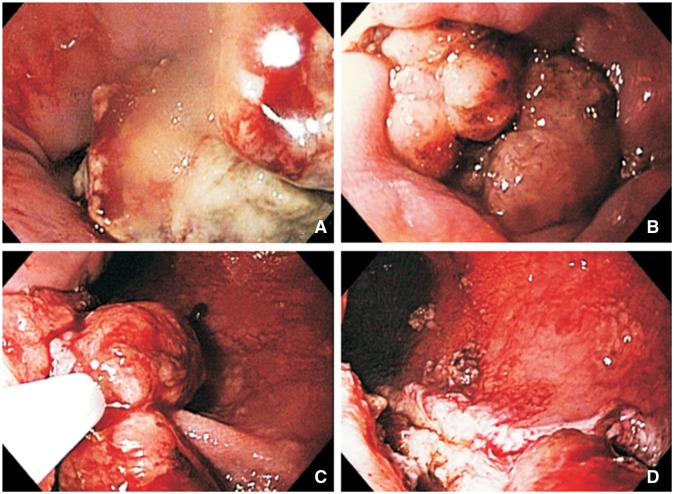
Endoscopic findings. (A) Lower esophagus showed two columns of tumor extending approximately 5 cm upstream from the gastroesophageal junction. (B) A bulky tumor causing a ball valve-like effect, obstructing the gastroesophageal junction. (C) Debulking was performed using a mini snare and a spiral snare. (D) Tumor burden was significantly decreased.

An 83-year-old Middle Eastern male with a medical history of meningioma and emphysema was admitted to our institution for evaluation of a liver mass. The mass had been found on an abdominal computed tomography (CT) scan conducted in an outside facility following the incidental finding of abnormal liver tests. A repeat abdominal CT scan revealed a large liver mass with central necrosis and extensive porta hepatis, periceliac and peripancreatic lymphadenopathy (Figure 1

). While laboratory tests showed only mild impairment of the liver tests, the AFP level was significantly elevated (>300 000 ng/mL; normal value <8.9 ng/mL). A CT-guided biopsy of the liver mass was performed. With cirrhosis not apparent, negative viral hepatitis serologies and the absence of any possible predisposing factors for HCC, the possibility of HCC was not deemed high. Therefore, while awaiting the pathology result of the liver mass, an esophagogastroduodenoscopy (EGD) was performed to explore the origin of this presumably metastatic lesion. The EGD revealed an esophageal mass adjacent to the gastroesophageal junction (Figure 2

**Figure 3.**
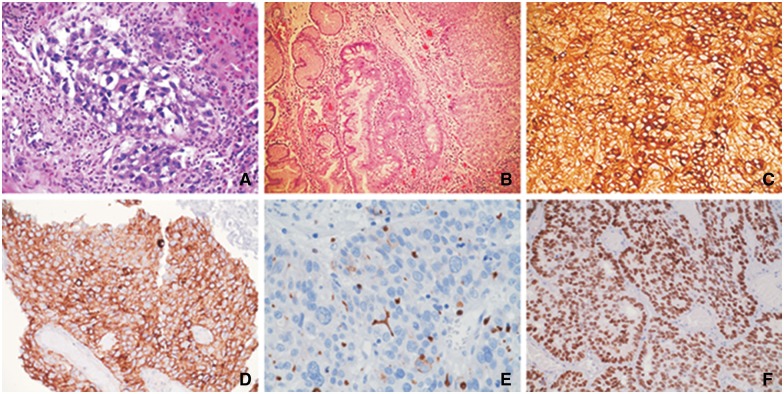
Histopathology and immunohistochemistry studies. (A) Liver specimens show polygonal tumor cells arranged mainly in a trabecular pattern and areas of glandular formations with abundant eosinophilic cytoplasm and round nuclei (hematoxylin and eosin stain, original magnification × 100). (B) Esophageal specimens show neoplastic hepatocyte-like cells arranged in nests and trabecular pattern with intervening fibrovascular stroma. Adjacent intestinal metaplasia (Barrett’s esophagus) is evident (hematoxylin and eosin stain, original magnification × 100). (C) Neoplastic cells from esophageal tumor were positive for alpha-fetoprotein staining (original magnification × 200). (D) Esophageal specimens show positive staining for glypican-3 (original magnification × 200). (E) Carcinoembryonic antigen (polyclonal) staining of esophageal specimens, although positive, did not show the definite canalicular staining pattern of hepatocellular carcinoma (original magnification × 400). (F) Esophageal specimens were positive for SALL4 staining (original magnification × 200).

A). Upon second review, the mass was seen on the abdominal CT scan that had been performed on admission. The results of both hepatic and esophageal lesion biopsies showed morphologically similar, poorly differentiated adenocarcinoma with hepatoid features, without the classic trabecular and sinusoidal pattern of HCC (Figure 3

A,B). The immunohistochemistry (IHC) studies did not show the distinctive pattern of HCC, cholangiocarcinoma or colorectal malignancies (Figure 3C–F). The specimens’ histologic and IHC characteristics were found to be consistent with AFP-producing HAC of the esophagus with liver metastasis.

**Figure 4.**
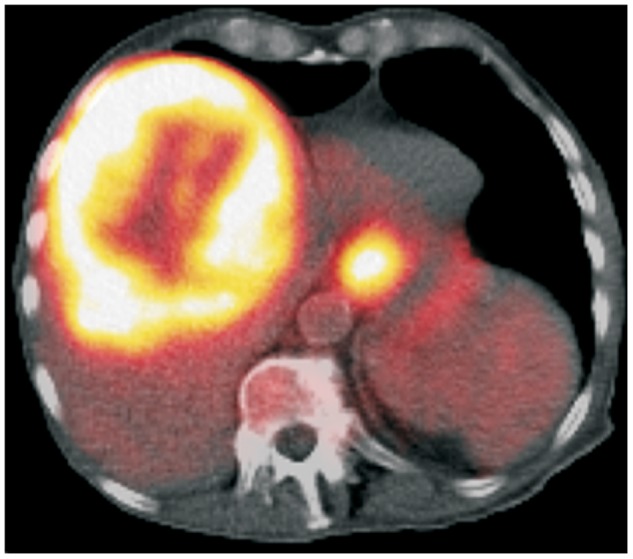
Positron emission/computed tomography scan reveals a large, intense metabolically active, low-density lesion with central photopenic area in the liver and an intense metabolically active area at the gastroesophageal junction.

In order to evaluate for other possible sites of metastases, an 18[F]-fluorodeoxyglucose positron emission tomography/CT scan was performed that showed the area of increased metabolic activities in the liver and esophagus (Figure 4

). Intense metabolically active areas, consistent with metastatic disease, were found in the left lung, mediastinum and intra-abdominal lymph nodes. As the patient was in generally poor health condition, aggressive therapy was not deemed beneficial. Two months later, the patient developed severe dysphagia. An EGD was repeated and showed that the tumor was obstructing the gastroesophageal junction (Figure 2B). Subsequent debulking was successfully performed (Figure 2C,D). Unfortunately, the patient expired a few days later, and autopsy was not permitted.

## Discussion

HAC is a subcategory of AFP-producing adenocarcinomas, which histologically mimic the architecture of HCC [3]. In animal studies, AFP-producing tumors with hepatoid features were more likely to metastasize to the liver compared with non-hepatoid types [5]. Most HAC cases originate from the gastric epithelium; other parts of the gastrointestinal tract, including the gallbladder and colon, are also reported as the origin of HAC [3].

A HAC arising from the esophagus is particularly rare. An extensive search of the literature produced only five reported cases in English-language journals and two cases in Japanese-language journals [4,6–11]. Although the limited number of cases makes it difficult to show significance, we analyzed the data from available cases including the current patient (Table 1

**Table 1.** Reported cases of esophageal hepatoid adenocarcinoma

	Motoyama *et al.* [6]	Tanigawa *et al.* [7]	Fukuzawa *et al.* [10]	Chiba *et al.* [8]	Atiq *et al.* [4]	Kuroda *et al.* [9]	Takeyama *et al.* [11]	Present patient
Country	Japan	Japan	Japan	Japan	USA^b^	Japan	Japan	USA^c^
Age (years)	80	44	55	47	56	76	58	83
Sex	Female	Female	Male	Male	Male	Male	Male	Male
Tumor location	LE	LE	LE/GE	LE	LE	LE	LE	LE/GE
AFP	NA	NL	47 800	326 400	>3000	N/A	3788	>300 000
Presentation	Poor appetite, fatigue	Poor appetite, chest discomfort	Dysphagia, hematemesis	Routine check-up	Fatigue, weight loss	Chest burn	Incidental	Abnormal liver tests
Barrett’s esophagus	Yes	Yes	No	Yes	No	Yes	Yes	Yes
Other tumors^a^	CHC, SCC, TAC	CHC, TAC	None	None	None	TAC	TAC	None
Metastasis	Liver, lung	Liver	Lung, bone, lymph node	Liver	Liver	N/A	None	Liver, lung, lymph node
Treatment	Bleomycin	Surgery, CTX	Surgery, CTX	Paclitaxel, Cisplatin	N/A	Surgery	Surgery, S-1	None
Survival (month)	2	4	9	14	N/A	3 (alive)	22 (alive)	4

AFP = alpha-fetoprotein (ng/mL); CHC = choriocarcinoma; CTX = chemotherapy (medications not specified); GE = gastroesophageal junction; LE = lower part of the esophagus; N/A = not available; SCC = small cell carcinoma; TAC = tubular adenocarcinoma.

^a^Coexistence of other tumors alongside hepatoid adenocarcinoma. ^b^Patient’s origin was unknown. ^c^Originally Middle Eastern.

). Patients with esophageal HAC generally present with constitutional symptoms including decreased appetite, weight loss and fatigue. The disease is more frequently found in the Asian population and in males. All cases have the tumor location in common; the lower part of the esophagus in proximity to the gastroesophageal junction appears to be the sole origin of the esophageal HAC. As in the present case, an association between esophageal HAC with Barrett’s esophagus has been reported [9]. However, it appears that this association is more prominent when HAC is found in combination with other histologic tumors such as tubular adenocarcinoma and choriocarcinoma. The tumor has an aggressive nature and in most cases presents with remote metastases at the time of diagnosis. The most common metastasis site is the liver, and patients generally present with an elevated AFP level. Therefore, esophageal HAC with liver metastasis in patients who primarily manifest with liver tumors and elevated AFP levels might be diagnostically confused with HCC. In these individuals, including the current patient, a medical history lacking cirrhosis or infection with chronic viral hepatitis favors the diagnosis of HAC.

Due to the histologic resemblance of HAC and HCC when histology is not typical of HCC and HAC is suspected, a meticulous diagnostic work-up is warranted to locate the origin of this presumably metastatic disease [3]. A pan-endoscopy of the GI tract including stomach, esophagus and colon should be performed, and appropriate liver imaging including dynamic CT scan or magnetic resonance imaging must be applied. The typical pattern of HCC on dynamic CT scan is contrast uptake in the arterial phase followed by “washout” in the venous phases; this pattern is not seen in metastatic liver lesions from HAC [8].

IHC studies are also found to be diagnostic. Su *et al.*, in a report on patients with HAC of different origins, showed that IHC staining of the epithelial markers cytokeratin 18, cytokeratin 19 and glypican-3 was positive in all tumors, and staining for AFP, cytokeratin AE1/AE3 and alfa-1 antitrypsin was positive in more than 90% of cases [3]. Different reports on HCC showed the expressions of glypican-3 in 84%, AE1/AE3 in 45%, AFP in 29%, cytokeratin 19 in 10%, and alfa-1 antitrypsin in 6% of cases [12–14]. So, in the presence of an AFP-producing liver lesion with hepatoid features, metastatic disease from HAC is highly suggested when staining for cytokeratin 19 or alfa-1 antitrypsin are positive. As in the current case, the presence of SALL4 seems promising for differentiating HAC from HCC. Expression of SALL4 has been found in 100% of AFP-producing gastric carcinomas, particularly in the hepatoid type, while HCC is negative for this staining in all cases [15]. Expression of MOC31 might be helpful when a liver lesion with hepatic features on pathology is suspected as a metastatic disease (e.g. lack of HCC risk factors) and a primary site is not found on initial work-up. MOC31 is only found in 12% of HCC cases, while it is positive in 66% of metastatic adenocarcinoma of the liver cases [13]. The IHC characteristics of reported esophageal HAC cases are shown in Table 2

**Table 2.** Immunohistochemistry characteristics of hepatoid adenocarcinoma of the esophagus among different reports

Antibody	Present patient		Motoyama *et al.* [6]	Tanigawa *et al.* [7]	Fukuzawa *et al.* [10]	Chiba *et al.* [8]	Atiq *et al.* [4]	Kuroda *et al.* [9]	Takeyama *et al.* [11]
	Liver	Esophagus							
Hep Par 1	–	N/A	N/A	–	N/A	+	+	+	N/A
CEA	+^a^	N/A	–	N/A	N/A	N/A	–	N/A	N/A
MOC31	+	N/A	N/A	N/A	N/A	N/A	N/A	N/A	N/A
AE1/AE3	+	+	N/A	N/A	N/A	N/A	+	N/A	N/A
AFP	+	+	+	+	+	+	+	+	+
Glypican-3	+	+	N/A	N/A	N/A	N/A	N/A	N/A	N/A
SALL4	N/A	+	N/A	N/A	N/A	N/A	N/A	N/A	N/A
A1AT	N/A	N/A	+	+	N/A	+	N/A	+	N/A
A1AC	N/A	N/A	N/A	N/A	N/A	N/A	N/A	+	N/A
Albumin	N/A	N/A	+	+	N/A	N/A	N/A	N/A	N/A
Prealbumin	N/A	N/A	+	–	N/A	N/A	N/A	N/A	N/A
Transferrin	N/A	N/A	+	–	N/A	N/A	N/A	N/A	N/A

A1AC = alpha-1 antichymotrypsin; A1AT = alfa-1 antitrypsin; AFP = alpha-fetoprotein; CEA = carcinoembryonic antigen; Hep Par 1 = hepatocyte paraffin 1; N/A = not available

^a^Although positive, did not show the definite canalicular staining pattern of hepatocellular carcinoma.

.

In conclusion, we have described a case of esophageal AFP-producing HAC that, although rare, stands as an important differential diagnosis of HCC. Where an AFP-producing neoplastic liver lesion with hepatoid features is identified—but without the typical characteristics of HCC on histology—lack of cirrhosis and other risk factors of HCC should raise the possibility of HAC. If metastatic disease is suspected, then extensive diagnostic work-up to identify the primary site is highly recommended. In cases with indeterminate histology, IHC studies might be quite helpful. Differentiation of HCC from HAC with liver metastasis is crucial due to the different clinicopathologic behaviors and therapeutic options for these entities.

## Authors’ contributions

Amir Kashani: conception and design; data collection; drafting of the article.

Jonathan C. Ellis: conception and design; data collection; critical revision of the article for important intellectual content.

Melissa Kahn: conception and design; data collection; critical revision of the article for important intellectual content.

Laith H. Jamil: conception and design; data collection, critical revision of the article for important intellectual content; final approval of the article.

*Conflict of interest statement*: none declared.
